# A Longitudinal Study of the Epidemiology of Seasonal Coronaviruses in an African Birth Cohort

**DOI:** 10.1093/jpids/piaa168

**Published:** 2021-02-02

**Authors:** Mark P Nicol, Rae MacGinty, Lesley Workman, Jacob A M Stadler, Landon Myer, Veronica Allen, Lemese Ah Tow Edries, Heather J Zar

**Affiliations:** 1 Division of Infection and Immunity, School of Biomedical Sciences, Faculty of Health Sciences, University of Western Australia, Perth, Australia; 2 Division of Medical Microbiology and Institute for Infectious Diseases and Molecular Medicine, University of Cape Town, Cape Town, South Africa; 3 Department of Paediatrics and Child Health and SA-MRC Unit on Child & Adolescent Health, Red Cross War Memorial Children’s Hospital and University of Cape Town, Cape Town, South Africa; 4 Division of Epidemiology and Biostatistics, School of Public Health and Family Medicine, University of Cape Town, Cape Town, South Africa

**Keywords:** children, co-infection, coronavirus, epidemiology, pneumonia

## Abstract

**Background:**

Since non-epidemic, seasonal human coronaviruses (sHCoV) commonly infect children, an improved understanding of the epidemiology of these infections may offer insights into the context of severe acute respiratory syndrome (SARS)-CoV-2. We investigated the epidemiology of sHCoV infection during the first year of life, including risk factors and association with lower respiratory tract infection (LRTI).

**Methods:**

We conducted a nested case-control study of infants enrolled in a birth cohort near Cape Town, South Africa, from 2012 to 2015. LRTI surveillance was implemented, and nasopharyngeal swabs were collected fortnightly over infancy. Quantitative PCR detected respiratory pathogens, including coronaviruses-229E, -NL63, -OC43, and -HKU1. Swabs were tested from infants at the time of LRTI and from the 90 days prior as well as from age-matched control infants from the cohort over the equivalent period.

**Results:**

In total, 885 infants were included, among whom 464 LRTI events occurred. Of the 4751 samples tested for sHCoV, 9% tested positive, with HCoV-NL63 the most common. Seasonal HCoV detection was associated with LRTI; this association was strongest for coronavirus-OC43, which was also found in all sHCoV-associated hospitalizations. Birth in winter was associated with sHCoV-LRTI, but there were no clear seasonal differences in detection. Co-detection of *Streptococcus pneumoniae* was weakly associated with sHCoV-LRTI (odds ratio: 1.8; 95% confidence interval: 0.9-3.6); detection of other respiratory viruses or bacteria was not associated with sHCoV status.

**Conclusions:**

Seasonal HCoV infections were common and associated with LRTI, particularly sHCoV-OC43, which is most closely related to the SARS group of coronaviruses. Interactions of coronaviruses with bacteria in the pathogenesis of LRTI require further study.

## Teaser

A longitudinal study of African infants showed that endemic coronavirus infections were common and weakly associated with lower respiratory tract infection (LRTI), particularly coronavirus-OC43, which was also associated with hospitalized LRTIs.

Human coronaviruses (HCoV) include the epidemic severe acute respiratory syndrome (SARS)-CoV-2, SARS-CoV, Middle East Respiratory Syndrome (MERS)-CoV, as well as 4 endemic, seasonal coronaviruses: 229E, NL63, OC43, and HKU1 [1]. These seasonal coronaviruses (hereafter referred to as sHCoV) circulate continually in human populations where they cause upper respiratory tract infections [2] or exacerbations of asthma [3]. Seasonal HCoV are also uncommon causes of lower respiratory tract infection (LRTI), predominately among immune-compromised individuals [4] or infants [5].

Symptomatic or severe illness appears to be uncommon in children following SARS-CoV-2 infection, with children accounting for only approximately 2% of those experiencing disease in the current coronavirus disease 2019 (COVID-19) pandemic [6, 7]. Children may nevertheless be important as reservoirs of infection or sources of transmission of SARS-CoV-2, but there are very limited data [8, 9]. Moreover, preexisting cross-reactive cellular immune responses to SARS-CoV-2, presumably due to prior infection with sHCoV, have been demonstrated and may afford some protection against COVID-19 [10, 11]. We, therefore, aimed to describe the incidence of and risk factors for sHCoV infection and the association between sHCoV and LRTI in an African birth cohort.

## METHODS

We conducted a nested case-control study of infants enrolled in a South African birth cohort, the Drakenstein Child Health Study [12], undertaken at 2 public primary health clinics in low-income communities in a peri-urban area near Cape Town. Pregnant women were enrolled during their second trimester, and infants followed from birth. All births occurred at a single hospital, from June 21, 2012, to August 12, 2015. Follow-up was at 6, 10, and 14 weeks and 6, 9, and 12 months. Where caregivers gave approval, a subset of infants (“intensive cohort”) was followed up fortnightly over the first year of life. Infants were given immunizations at primary care clinics including 4 doses of a 5 vaccine combination (diphtheria, tetanus, acellular pertussis, *Haemophilus influenzae* type b, and inactivated polio vaccine) at 6, 10, and 14 weeks and 18 months; measles vaccine at 9 months and 18 months; and 13-valent pneumococcal conjugate vaccine at 6 weeks, 14 weeks, and 9 months.

LRTI or pneumonia surveillance took place at local clinics and the nearby district hospital [13]. Mothers were counseled regarding respiratory symptoms and advised to contact study staff whenever symptoms developed. Primary healthcare nurses and study staff were trained to recognize the World Health Organization (WHO)-defined pneumonia. Measurement of risk factors (nutrition, environment, vaccinations received, and child and maternal factors) and anthropometry were carried out at each study visit, as described previously [12], including maternal smoking or passive smoke exposure measured by self-report.

Infants were eligible for inclusion in this analysis if they were part of the intensive cohort (nasopharynx sampled fortnightly). LRTI cases were defined as clinical episodes meeting the WHO case definition for pneumonia or severe pneumonia during the first year of life, excluding congenital episodes (occurring prior to discharge from hospital after birth). Controls (without WHO-defined pneumonia, but who may have had other respiratory symptoms) were incidence-density matched to cases (1:1) by birth date (to within 2 weeks), age of presentation (to within 2 weeks), and site of enrollment. Controls could be sampled more than once at different ages, but this occurred infrequently.

Ethics approval was obtained from the University of Cape Town, Faculty of Health Sciences Research Ethics Committee. Mothers provided written informed consent.

### Microbiological Sampling and Testing

Nasopharyngeal swabs were collected fortnightly over infancy for the intensive cohort. In addition, all infants had nasopharyngeal swabs taken at the time of LRTI. In a subset of infants with LRTI, swabs could not be collected due to the unavailability of study staff at the time of presentation. Nasopharyngeal swabs were transferred into nucleic acid preservation medium (PrimeStore, Longhorn Vaccines and Diagnostics, San Antonio, TX, USA), transported on ice, and frozen at –80°C for batch testing.

We tested swabs from the time of LRTI or the matched time point in controls, as well as all available swabs collected in the 90 days preceding the LRTI event (or the matched time period for controls). Swabs underwent mechanical lysis on a Tissuelyzer LT (Qiagen, Hilden, Germany) followed by total nucleic acid extraction (QIAsymphony Virus/Bacteria Mini Kit, Qiagen, Hilden, Germany). Quantitative, multiplex, real-time PCR (qPCR) with FTDResp33 (Fast-Track Diagnostics, Esch-sur-Alzet, Luxembourg) identified potential respiratory pathogens including respiratory viruses (HCoV-NL63, -229E, -OC43, -HKU1; influenza A, B, and C; parainfluenza 1, 2, 3, and 4; human metapneumoviruses A and B; rhinovirus; respiratory syncytial viruses A and B; adenovirus; enterovirus; parechovirus; bocavirus; and cytomegalovirus) and bacteria (*Streptococcus pneumoniae*, *H. influenza, Staphylococcus aureus*, and *Moraxella catarrhalis*). Standard curves were derived using standards supplied by the manufacturer.

### Analysis

The sHCoV result was defined as positive if any of HCoV-229E, -NL63, -OC43, and -HKU1 were positive by qPCR. LRTI episodes were stratified into those in whom the swab collected at LRTI was positive for any of the 4 sHCoV (sHCoV+ cases) and those in whom the swab was negative for all sHCoVs (sHCoV− cases). Controls were similarly stratified based on whether the swab collected at the time point corresponding to LRTI in the matched case tested positive or negative for any sHCoV (sHCoV+ controls or sHCoV− controls).

Analysis of risk factors was done on a per-child basis [14]. In order to account for intraindividual clustering, we used generalized estimating equation (GEE) models to explore the risk factors for LRTI by the sHCoV subgroup. In each of the GEE models, a binomial distribution was considered with a logit link function. Data were analyzed using STATA 15.1 (STATA Corporation, College Station, TX, USA). Weight-for-age at birth *Z*-scores were derived from the Fenton’s growth standards [15]. Socioeconomic status was evaluated in quartiles [12]. Prematurity was defined as birth at <37 weeks of gestation.

All statistical tests were 2-sided at α = 0.05. Kruskal Wallace, Wilcoxon rank-sum test, Chi-square, or Fisher’s exact was used for crude comparison, as appropriate. Child follow-up over the 90-day period of sampling was used to calculate person-time at risk, with a not at risk period of 2 weeks after an LRTI.

Acquisition of sHCoV was defined by first sHCoV+ sample, a change of sHCoV species, and a sHCoV+ test following 2 consecutive sHCoV− tests or more than 2 months after the last positive test. The acquisition was assumed to start at the midpoint between the last of 2 negative consecutive tests (missed visits were regarded as having negative tests) and the first sHCoV+ test. If the first swab collected for a child was sHCoV+, the acquisition was assumed to start 7 days prior. Clearance was assumed to be the midpoint between the last sHCoV+ sample and the first of 2 consecutive negative (or missing) results. If carriage extended past the final sample collected, the acquisition was assumed to end in 7 days after the final sample collection date. The duration between acquisition and clearance was considered a carriage episode.

## RESULTS

**Figure 1.**
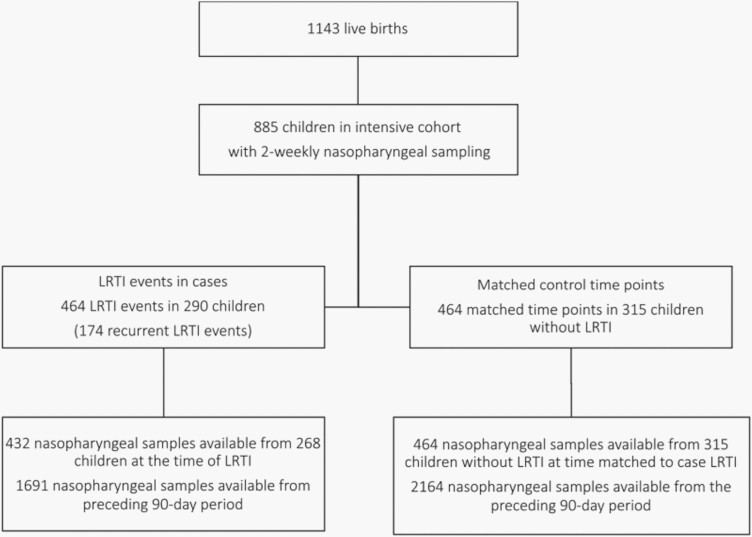
Study profile showing the number of included children, episodes of lower respiratory tract infection, and matched control time points and corresponding nasopharyngeal samples.

Overall, 1143 live births occurred in 1137 mothers, of whom 885 (77%) were enrolled into the intensive cohort with 2 weekly nasopharyngeal swab collection (Figure 1). Cohort retention was 89% at 1 year. Among the 885 infants in the intensive cohort, there were a total of 464 LRTI events in 290 infants over the first year of life (174 LRTI events were recurrent), which were matched to 464 equivalent time points from 315 control infants. From the 464 LRTI events, 432 nasopharyngeal samples were available (samples were not available from 32 events), from 268 infants at the time of LRTI. An additional 1691 samples were available from LRTI cases from the period 90 days preceding LRTI, while 2164 samples were available from controls over the equivalent period. More samples were collected in autumn (1484 [31%]) and winter (1500 [32%]) than from spring (959 [20%]) or summer (808 [17%]).

Characteristics of infants experiencing LRTI (cases) and matched controls, stratified by sHCoV status, are given in Table 1. Eighteen percent of infants were born prematurely, 27% were HIV exposed, but all were HIV uninfected at birth. The prevalence of maternal smoking in pregnancy was 25%. Duration of exclusive breastfeeding was a median (interquartile range [IQR]) of 1.4 (0.5-3.2) months. Median ages were similar among cases and controls, irrespective of sHCoV status. Vaccine coverage was excellent: 100% for the primary series and 99% at 9 months.

**Table 1.** Demographic and Clinical Characteristics of Children With LRTI (Cases) and Matched Controls, by sHCoV Results

Measure	All Episodes N (%)	sHCoV+ Cases N (%)	sHCoV− Cases N (%)	sHCoV+ Controls N (%)	sHCoV− Controls N (%)	*P*-value
Total number (episodes)	896	46 (5)	386 (43)	38 (4)	426 (48)	
Child characteristics at birth						
Male, n (%)	491 (55)	27 (59)	237 (61)	18 (47)	209 (49)	.004
Premature (<37 weeks’ gestation)	164 (18)	8 (17)	92 (24)	5 (13)	59 (14)	.003
Birth weight-for-age *Z*-score	−0.6 (−1.3; 0.1)	−0.5 (−1.3; 0.0)	−0.8 (−1.4; 0.0)	−0.3 (−1.2; 0.6)	−0.4 (−1.2; 0.2)	.030
HIV exposed	241 (27)	12 (26)	120 (31)	6 (16)	103 (24)	.059
Season of birth						
Winter	215 (24)	18 (39)	83 (22)	8 (21)	106 (25)	.206
Spring	201 (22)	12 (26)	95 (25)	9 (24)	85 (20)	
Summer	217 (24)	7 (15)	90 (23)	11 (29)	109 (26)	
Autumn	263 (29)	9 (20)	118 (31)	10 (26)	126 (30)	
Child characteristics at episode (cases) or matched reference time (controls)						
Age months, median (IQR)	4.6 (2.8-7.4)	4.6 (2.8-7.0)	4.7 (2.8-7.6)	4.6 (3.1-6.8)	4.6 (2.8-7.4)	.988
Season of episode/reference						
Winter	260 (29)	11 (24)	108 (28)	131 (31)	10 (26)	.700
Spring	259 (29)	17 (37)	113 (29)	119 (28)	10 (26)	
Summer	132 (15)	10 (22)	57 (15)	58 (14)	7 (18)	
Autumn	245 (27)	8 (17)	108 (28)	118 (28)	11 (29)	
Maternal characteristics at birth						
Maternal smoking during pregnancy (self-report)	225 (25)	10 (22)	97 (25)	7 (18)	111 (26)	.709
Socioeconomic quartiles						
Low	231 (26)	17 (37)	101 (26)	9 (24)	104 (24)	<.001
Low-moderate	250 (28)	10 (22)	141 (37)	8 (21)	91 (21)	
Moderate-high	213 (24)	7 (15)	85 (22)	9 (24)	112 (26)	
High	202 (23)	12 (26)	59 (15)	12 (32)	119 (28)	
Duration exclusive breastfeeding						
In months	1.4 (0.5-3.2)^a^	1.8 (0.5-3.7)	1.1 (0.5-2.8)	1.6 (0.9-3.3)	1.6 (0.7-3.2)	.136

Analyses presented on a per-episode basis.

Abbreviations: LRTI, lower respiratory tract infection; IQR, interquartile range; nHCoV+, defined as positive if positive for any of the 4 endemic coronaviruses.

^a^889/896 (99%) data available for duration in months of exclusive breastfeeding.

### Comparison of sHCoV+ and sHCoV− Cases and Controls at the Time of LRTI

Among the LRTI events, 46 of the 432 (10.6%) cases tested positive for any sHCoV at the time of LRTI (sHCoV+ cases), while, among the controls, 38 of the 464 (8.2%) cases tested positive at the matched time point (sHCoV+ controls) *P* = .207 (Table 2).

**Table 2.** Prevalence of Positive sHCoV-Positive Tests, at the Time of LRTI as well as in the 90 Days Preceding LRTI

	All Episodes n (%, 95% CI)		Cases n (%, 95% CI)		Controls n (%, 95% CI)	
	Time of LRTI/Matched	Preceding 90 Days	Time of LRTI	Preceding 90 Days	Matched to Time of LRTI	Preceding 90 Days
N tested	896	3855	432	1691	464	2164
Any sHCoV+	84 (9.4, 7.5-11.4)	342 (8.9, 8.0-9.8)	46 (10.6, 7.9-13.9)	155 (9.2, 7.8-10.6)	38 (8.2, 5.9-11.1)	187 (8.6, 7.5-9.9)
HCoV-NL63+	22 (2.5, 1.5-3.7)	124 (3.2, 2.7-3.8)	11 (2.5, 1.3-4.5)	53 (3.1, 2.4-4.1)	11 (2.4, 1.2-4.2)	71 (3.3, 2.6-4.1)
HCoV-229E+	5 (0.6, 0.2-1.3)	17 (0.4, 0.3-0.7)	4 (0.9, 0.3-2.4)	10 (0.6, 0.3-1.1)	1 (0.2, 0.01-1.2)	7 (0.3, 0.1-0.7)
HCoV-OC43+	38 (4.2, 3.0-5.8)	98 (2.5, 2.1-3.1)	24 (5.6, 3.6-8.2)	52 (3.1, 2.3-4.0)	14 (3.0, 1.7-5.0)	46 (2.1, 1.6-2.8)
HCoV-HKU1+	20 (2.2, 1.4-3.4)	110 (2.9, 2.4-3.4)	8 (1.9, 0.8-3.6)	43 (2.5, 1.8-3.4)	12 (2.6, 1.3-4.5)	67 (3.1, 2.4-3.9)

Abbreviations: CI, confidence interval; sHCoV, seasonal human coronavirus.

We used GEE to explore the risk factors for LRTI using 3 models: model A: all cases vs all controls; model B: sHCoV+ cases vs sHCoV+ controls; and model C: sHCoV+ cases vs sHCoV− cases (Table 3). Cases were more likely to be male, preterm, have low birth for weight *Z*-scores, and from low socioeconomic quartiles than controls (model A). In addition, LRTI cases were more likely to test positive for any sHCoV (adjusted odds ratio [OR]: 1.8; 95% confidence interval [CI]: 1.2-2.6), HCoV-OC43 (unadjusted OR: 2.5; 95% CI: 1.4-4.2), and HCoV-229E (unadjusted OR: 2.8; 95% CI: 0.6-14.4) contributed most to this effect.

**Table 3.** Results of Multivariate Modeling of Risk Factors Associated With Any LRTI (Model A), LRTI Among sHCoV+ Children (Model B), or sHCoV− Status Among LRTI Cases (Model C)

	Model A: All Cases vs all Controls		Model B: sHCoV+ Cases vs sHCoV+ Controls	Model C: sHCoV+ Cases vs sHCoV− Cases
	Unadjusted OR (95% CI)	Adjusted OR^a^ (95% CI)	Adjusted OR (95% CI)	Adjusted OR (95% CI)
sHCoV detection at the time of LRTI				
Any sHCoV	1.5 (1.1-2.1)	1.8 (1.2-2.6)^b^		
HCoV-NL63	1.1 (0.6-2.0)			
HCoV-229E	2.8 (0.6-14.4)			
HCoV-OC43	2.5 (1.4-4.2)			
HCoV-HKU1	1.1 (0.5-2.1)			
Other pathogen detection at the time of LRTI				
RSV	3.0 (2.1-4.3)	4.2 (2.8-6.2)		
Influenzae viruses A & B	2.8 (1.3-6.3)	3.9 (1.6-9.2)		
Parainfluenza viruses 1, 2, 3, and 4	1.6 (1.1-2.5)	1.9 (1.2-3.1)		
*H. influenza*	1.4 (1.1-1.7)	1.3 (1.0-1.7)		
Metapneumovirus A & B	1.6 (1.0-2.4)	1.8 (1.1-2.8)		
Child characteristics at birth				
Sex: Male (ref. female)	1.6 (1.1-2.2)	1.6 (1.1-2.3)	2.4 (0.7-7.9)	0.9 (0.5-1.9)
Preterm	1.8 (1.1-2.7)	2.1 (1.3-3.5)	4.0 (0.8-20.6)	0.7 (0.3-1.8)
Birth weight-for-age *Z*-score	0.9 (0.8-1.1)	0.8 (0.7-1.0)	0.7 (0.4-1.1)	1.1 (0.8-1.5)
Season of birth (ref. summer)				
Autumn (Mar-May)	1.1 (0.7-1.8)	1.1 (0.7-1.8)	1.2 (0.3-5.6)	1.0 (0.3-2.9)
Winter (Jun-Aug)	1.1 (0.7-1.7)	1.1 (0.7-1.8)	5.3 (1.1-25.0)	2.8 (1.1-7.3)
Spring (Sep-Nov)	1.2 (0.7-1.8)	1.3 (0.8-2.1)	1.5 (0.3-6.7)	1.4 (0.5-4.2)
HIV exposed	1.4 (1.0-2.0)	1.2 (0.8-1.8)	1.5 (0.4-6.3)	0.7 (0.3-1.6)
Child characteristics at LRTI/matched time				
Age category (ref. 0-3 months)				
3-6 months	1.0 (0.8-1.3)	0.9 (0.7-1.2)	0.7 (0.2-2.3)	0.8 (0.4-1.7)
6-12 months	1.1 (0.8-1.5)	1.0 (0.7-1.3)	1.3 (0.4-4.2)	0.8 (0.4-1.7)
Maternal characteristics				
Antenatal cigarette smoking	0.9 (0.6-1.3)	0.9 (0.6-1.4)	1.1 (0.3-4.2)	0.8 (0.3-1.7)
Socio economic quartile (ref. high)				
Low	1.5 (0.9-2.4)	1.4 (0.8-2.2)	1.9 (0.5-7.6)	0.9 (0.4-2.3)
Low-moderate	2.4 (1.5-3.8)	2.2 (1.3-3.6)	1.4 (0.3-6.1)	0.5 (0.2-1.2)
Moderate-high	1.3 (0.8-2.0)	1.2 (0.7-2.1)	1.0 (0.2-5.0)	0.5 (0.2-1.4)
Exclusive breastfeeding (months)	1.0 (0.9-1.0)	1.0 (0.9-1.1)	0.9 (0.7-1.3)	1.1 (0.9-1.3)

Abbreviations: LRTI, lower respiratory tract infection; OR, odds ratio; 95% CI, 95% confidence interval; nHCoV+, defined as positive if positive for any of the 4 endemic coronaviruses; sHCoV, seasonal human coronavirus.

^a^Adjusted for all covariates in the table.

^b^Only one sHCoV covariate could be included simultaneously in the model.

**Figure 2.**
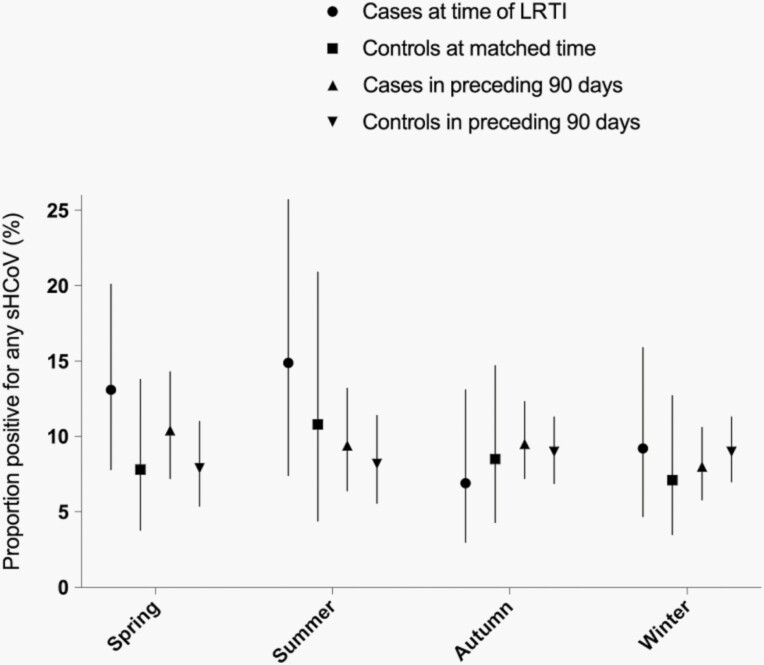
The proportion of samples tested, stratified by season of sample collection and case or control status, which were positive for any seasonal human coronaviruses.

Risk factors for LRTI among participants who were sHCoV+ (model B) were similar to those identified when comparing all cases with all controls. However, sHCoV+ cases were more likely to have been born in winter than sHCoV+ controls (adjusted OR: 5.3; 95% CI: 1.1-25.0) or sHCoV− cases (adjusted OR: 2.8; 95% CI: 1.1-7.3). Season of the episode could not be evaluated in the GEE models together with season of birth, but the distribution of participants by category was similar (Table 1). We further explored the proportion of infants with positive sHCoV tests by season (Figure 2). Among infants with LRTI, the proportion with a positive test for sHCoV was higher in spring and summer (13.7%) than autumn and winter (8.1%); however, this difference was not statistically significant, after adjusting for multiple episodes per participant (*P* = .065).

The symptoms of sHCoV+ cases and sHCoV− cases were similar, with cough, blocked nose, and diarrhea nonsignificantly more common in sHCoV+ cases. Seasonal HCoV+ cases were not more likely to be hospitalized (8/46, 17%) than sHCoV− cases (98/386, 25%) (Supplementary Table 1). Since HCoV-OC43 was most clearly associated with LRTI, we compared the clinical presentation of cases positive for HCoV-OC43 with cases positive for the other sHCoVs (Supplementary Table 2). Strikingly, all 8 infants hospitalized with LRTI who had a positive test for sHCoV were HCoV-OC43 positive.

Among controls, there was no difference in the prevalence of symptoms of upper respiratory tract infection at the time of specimen collection between sHCoV+ controls and sHCoV− controls (Supplementary Table 3). The majority (61%) of sHCoV+ controls had no symptoms. There was a small but significant difference in the viral load between infants (cases or controls) with symptoms (6.6 log_10_ copies/mL) and those without symptoms (6.3 log_10_ copies/mL), *P* = .03.

### Comparison of sHCoV+ and sHCoV− Cases and Controls Over the 90 Days Preceding LRTI

Of all of the 4751 samples tested for sHCoV (samples were taken at the time of LRTI as well as in the preceding 90 days or matched period for controls), 426 (9.0%) tested positive (Table 2). HCoV-NL63 was the most common (146/426, 34.3%) followed by -OC43 (136/426, 31.9%), -HKU1 (130/426, 30.5%), and -229E (22/426, 5.2%).

The incidence of sHCoV acquisition (when consecutive positive results for the same sHCoV were regarded as a single acquisition event) among cases and controls was similar: 2.05 events per child year (95% CI: 1.74-2.41) and 1.63 events per child year (95% CI: 1.39-1.91), respectively (Table 4), although the incidence of acquisition of HCoV-OC43 and HCoV-229E was significantly higher for cases. The median duration of detection of sHCoV was similar between cases and controls (median [IQR] of 15 days [14–24] and 16 days [14–28], respectively).

**Table 4.** Number of sHCoV Acquisitions and Incidence of sHCoV Acquisition in Cases vs Controls

	Total		Cases		Controls		Odds Ratio
	sHCoV Acquisitions N	Incidence Rate^a^ (95% CI)	sHCoV Acquisitions N	Incidence Rate^b^ (95% CI)	sHCoV Acquisitions N	Incidence Rate^c^ (95% CI)	Cases vs Controls (95% CI)
Any nHCoV	294	1.81 (1.62-2.03)	146	2.05 (1.74-2.41)	148	1.63 (1.39; 1.91)	1.18 (1.03-1.36)
HCoV-NL63	99	0.61 (0.50-0.74)	47	0.66 (0.50-0.88)	52	0.57 (0.44; 0.75)	1.18 (0.94-1.48)
HCoV-229E	16	0.10 (0.06-0.16)	11	0.15 (0.09-0.28)	5	0.06 (0.02; 0.13)	2.25 (1.23-4.12)
HCoV-OC43	97	0.60 (0.49-0.73)	53	0.74 (057-0.97)	44	0.48 (0.36; 0.65)	1.34 (1.06-1.70)
HCoV-HKU1	82	0.51 (0.41-0.63)	35	0.49 (0.35-0.68)	47	0.52 (0.39; 0.69)	0.92 (0.71-1.19)

Abbreviations: CI, confidence interval; sHCoV, seasonal human coronavirus.

^a^Total risk time = 59 201 days/162.08 years.

^b^Total risk time = 26 028 days/71.26 years.

^c^Total risk time = 33 173 days/90.82 years.

The viral load (measured by quantitative PCR) of sHCoV in the nasopharynx did not change in the period leading up to LRTI and was not substantially different between infants hospitalized with LRTI, infants with ambulatory LRTI, and controls (Supplementary Figure 1).

Sequential infection, or coinfection, with different sHCoV over the 90-day interval was observed in 16 infants (9 cases and 7 controls), of whom 8 had detection of 2 different sHCoVs in the same sample.

The detection of nasopharyngeal commensal bacteria (*S. pneumoniae*, *S. aureus*, *H. influenzae*, or *M. catarrhalis*) was similar when comparing cases and controls, except for *H. influenzae*, which was more likely to be detected in cases (Table 5). There were no differences in the detection of commensals between sHCoV+ cases and sHCoV− cases, except the odds of detecting *S. pneumoniae* in sHCoV+ cases vs sHCoV− cases were 1.8 (95% CI: 0.9-3.6). There were no differences in bacterial load for each of the 4 bacterial species, between cases and controls or in relation to the sHCoV status (Supplementary Figure 2).

**Table 5.** Results of Multivariate Modeling of Bacterial Pathogens Associated With Any LRTI (Model A) and sHCoV-Associated LRTI (Model B)

	Model A: All Cases vs All Controls	Model B: sHCoV+ Cases vs sHCoV− Cases
	Adjusted OR (95% CI)	Adjusted OR (95% CI)
*Staphylococcus aureus*	1.1 (0.8-1.4)	1.0 (0.4-2.2)
*Streptococcus pneumoniae*	1.1 (0.9-1.4)	1.8 (0.9-3.6)
*Moraxella catarrhalis*	1.1 (0.9-1.5)	1.4 (0.7-3.1)
*Haemophilus influenzae*	1.3 (1.0-1.7)	1.1 (0.6-2.1)

Abbreviations: OR, odds ratio; 95% CI, 95% confidence interval; nHCoV+, defined as positive if positive for any of the 4 endemic coronaviruses. Models adjusted for age at episode/reference.

Rhinovirus and cytomegalovirus were commonly co-detected with sHCoV (Supplementary Table 4). While RSV, influenza A and B, parainfluenza viruses, and adenovirus were all more commonly detected in cases compared with controls, there were no significant differences in the detection of any viruses in sHCoV+ cases compared with sHCoV− cases.

## DISCUSSION

Our knowledge of the epidemiology of sHCoV has largely been derived from case series or from cross-sectional studies of pneumonia etiology. Here, we present detailed data on the epidemiology of sHCoV infections in infants participating in a birth cohort in an African population. Seasonal HCoV infection was common among infants followed through the first year of life (1.63 and 2.05 events per child year among controls and cases, respectively).

There was a weak but significant association between sHCoV and LRTI, consistent with the literature that these viruses uncommonly cause LRTI in immunocompetent children [16]. However, this varied between species, with HCoV-OC43 more strongly associated with LRTI, including hospitalized LRTI. HCoV-OC43 is phylogenetically the most closely related of the sHCoV to the SARS group of coronaviruses [17]. The number of cases with HCoV-229E was small, precluding the definitive assessment of association with LRTI; however, the suggestive results indicate the need for more studies.

The risk factors for sHCoV-associated LRTI were similar to those for all LRTI. However, while there was no seasonal pattern to sHCoV infection, birth in winter was significantly associated with sHCoV detection among cases and with the presence of LRTI among infants positive for sHCoV. Season of birth has previously been associated with poor birth outcomes [18] and nutritional indicators in children [19, 20], which may be related to dietary or environmental factors [19]. Children born in autumn or winter have an increased risk of wheezing or asthma in later life, which may be partly mediated by respiratory tract infection [21].

In 16 infants, we identified coinfection with more than one sHCoV simultaneously, or within short intervals of each other, suggesting that immunity against one sHCoV does not give complete cross protection against other sHCoV species.

There were no symptoms or signs that distinguished children infected with sHCoV among cases. The majority of sHCoV+ controls were asymptomatic. This is consistent with reports of seasonal coronavirus in children that have described nonspecific symptoms or signs [6]. However, in our study, the presence of symptoms was associated with higher viral load, and infants with HCoV-OC43-associated LRTI had more severe illness, requiring hospitalization. The apparent low pathogenicity of sHCoV does not allow us to draw any similar conclusions about SARS-CoV-2.

Since sHCoV status and sHCoV viral load were only weakly associated with LRTI, it is probable that LRTI in many sHCoV+ cases was due to other pathogens. Indeed, viruses that are significantly associated with LRTI, such as RSV, influenza, and parainfluenza viruses, were frequently detected. There was no significant association between other viruses and the presence of sHCoV in cases.

Data from in vitro and animal studies suggest that interactions between human coronaviruses and bacteria may be important. HCoV-NL63 infection enhances the adherence of *S. pneumoniae* to primary human epithelium cultures [22]. Lipoteichoic acid, from the cell wall of *S. aureus*, stimulates the secretion of proinflammatory cytokines, which increased susceptibility to coronavirus in a swine model [23]. We, therefore, explored co-detection with bacteria. We identified a potential association between *S. pneumoniae* and sHCoV infection among cases (OR: 1.8; 95% CI: 0.9-3.6), which requires further study. Interactions between *S. pneumoniae* and influenza A virus are thought to have been important in the 1918 influenza pandemic [24], while co-detection of *S. pneumoniae* with RSV has been associated with increased clinical severity compared with detection of RSV alone [25].

Limitations of our study which might affect generalizability are the use of only 2 study sites; however, these sites are broadly representative of many childhood populations in low- and middle-income countries. Only the first year of life was studied; however, this is the highest risk period for LRTI in children. Since we only tested samples from the 90 days prior to LRTI event, we may have underestimated the duration of carriage. We used the WHO definition of pneumonia, which is based on clinical symptoms alone, and may lack specificity. Strengths include detailed longitudinal sampling, testing for a wide range of LRTI pathogens, measurements of a range of risk factors, and the community-based cohort study with high cohort retention.

In summary, sHCoV infections were common in infants in this setting and associated with LRTI, particularly HCoV-OC43. Seasonality of birth was associated with sHCoV+ LRTI. Our finding in relation to the co-detection of *S. pneumoniae* suggests that interactions of human coronaviruses with bacteria in the pathogenesis of LRTI require further study.

## Supplementary Data

Supplementary materials are available at the *Journal of the Pediatric Infectious Diseases Society* online (http://jpids.oxfordjournals.org).

piaa168_suppl_Supplementary_Figure_1

piaa168_suppl_Supplementary_Figure_2

## Notes

***Acknowledgments***. We would like to thank the children and families participating in the Drakenstein Child Health Study. We thank the study staff and also the clinical and administrative staff of the Western Cape Government Health Department at Paarl Hospital, TC Newman, and Mbekweni clinics for their support of the study.

***Financial support.*** This work was supported by the National Institutes of Health Common Fund, through the Office of Strategic Coordination/Office of the NIH Director, National Institute of Environmental Health Sciences and National Human Genome Institute of Health of the National Institutes of Health (H3Africa awards grant numbers U54HG009824, 1U01AI110466) and Bill & Melinda Gates Foundation, Seattle, WA (grant numbers OPP1017641, OPP1017579]. M. P. N. is supported by an Australian National Health and Medical Research Council Investigator Grant (grant number APP1174455). The funding bodies had no role in the study design, the collection, analysis, or interpretation of the data or the writing of the manuscript.

***Potential conflicts of interest***. All authors: No reported conflicts.

## References

[1] Corman VM , Muth D, Niemeyer D, Drosten C. Hosts and sources of endemic human coronaviruses. Adv Virus Res 2018; 100:163–88. 29551135 10.1016/bs.aivir.2018.01.001 PMC7112090

[2] van Elden LJ , van Loon AM, van Alphen F, et al Frequent detection of human coronaviruses in clinical specimens from patients with respiratory tract infection by use of a novel real-time reverse-transcriptase polymerase chain reaction. J Infect Dis 2004; 189:652–7. 14767819 10.1086/381207 PMC7110206

[3] Zheng XY , Xu YJ, Guan WJ, Lin LF. Regional, age and respiratory-secretion-specific prevalence of respiratory viruses associated with asthma exacerbation: a literature review. Arch Virol 2018; 163:845–53. 29327237 10.1007/s00705-017-3700-y PMC7087223

[4] Mayer K , Nellessen C, Hahn-Ast C, et al Fatal outcome of human coronavirus NL63 infection despite successful viral elimination by IFN-alpha in a patient with newly diagnosed ALL. Eur J Haematol 2016; 97:208–10. 26854965 10.1111/ejh.12744 PMC7163643

[5] Konca C , Korukluoglu G, Tekin M, et al The first infant death associated with human coronavirus NL63 infection. Pediatr Infect Dis J 2017; 36:231–3. 28081049 10.1097/INF.0000000000001390

[6] Ludvigsson JF . Systematic review of COVID-19 in children shows milder cases and a better prognosis than adults. Acta Paediatr 2020; 109:1088–95. 32202343 10.1111/apa.15270 PMC7228328

[7] Tagarro A , Epalza C, Santos M, et al. Screening and severity of coronavirus disease 2019 (COVID-19) in Children in Madrid, Spain. JAMA Pediatr 2020. doi:10.1001/jamapediatrics.2020.1346 PMC7142799 32267485

[8] Park YJ , Choe YJ, Park O, et al; COVID-19 National Emergency Response Center, Epidemiology and Case Management Team. Contact tracing during coronavirus disease outbreak, South Korea, 2020. Emerg Infect Dis 2020; 26:2465–8. 32673193 10.3201/eid2610.201315 PMC7510731

[9] Kim L , Whitaker M, O’Halloran A, et al; COVID-NET Surveillance Team. Hospitalization rates and characteristics of children aged <18 years hospitalized with laboratory-confirmed COVID-19 – COVID-NET, 14 States, March 1-July 25, 2020. MMWR Morb Mortal Wkly Rep 2020; 69:1081–8. 32790664 10.15585/mmwr.mm6932e3 PMC7440125

[10] Grifoni A , Weiskopf D, Ramirez SI, et al Targets of T cell responses to SARS-CoV-2 coronavirus in humans with COVID-19 disease and unexposed individuals. Cell 2020; 181:1489–501.e15. 32473127 10.1016/j.cell.2020.05.015 PMC7237901

[11] Mateus J , Grifoni A, Tarke A, et al Selective and cross-reactive SARS-CoV-2 T cell epitopes in unexposed humans. Science 2020; 370:89–94. 32753554 10.1126/science.abd3871 PMC7574914

[12] Zar HJ , Barnett W, Myer L, et al Investigating the early-life determinants of illness in Africa: the Drakenstein Child Health Study. Thorax 2015; 70:592–4. 25228292 10.1136/thoraxjnl-2014-206242 PMC5107608

[13] le Roux DM , Myer L, Nicol MP, Zar HJ. Incidence of childhood pneumonia: facility-based surveillance estimate compared to measured incidence in a South African birth cohort study. BMJ Open 2015; 5:e009111. 10.1136/bmjopen-2015-009111 PMC4691755 26685027

[14] Zar HJ , Barnett W, Stadler A, et al Aetiology of childhood pneumonia in a well vaccinated South African birth cohort: a nested case-control study of the Drakenstein Child Health Study. Lancet Respir Med 2016; 4:463–72. 27117547 10.1016/S2213-2600(16)00096-5 PMC4989125

[15] Group WHOMGRS. WHO Child Growth Standards based on length/height, weight and age. Acta Paediatr Suppl 2006; 450:76–85. 16817681 10.1111/j.1651-2227.2006.tb02378.x

[16] Ogimi C , Kim YJ, Martin ET, et al What’s new with the old coronaviruses? J Pediatric Infect Dis Soc 2020; 9:210–7. 32314790 10.1093/jpids/piaa037 PMC7188130

[17] Coronaviridae Study Group of the International Committee on Taxonomy of Viruses. The species severe acute respiratory syndrome-related coronavirus: classifying 2019-nCoV and naming it SARS-CoV-2. Nat Microbiol 2020; 5:536–44. 32123347 10.1038/s41564-020-0695-z PMC7095448

[18] Hughes MM , Katz J, Mullany LC, et al Seasonality of birth outcomes in rural Sarlahi District, Nepal: a population-based prospective cohort. BMC Pregnancy Childbirth 2014; 14:310. 25195204 10.1186/1471-2393-14-310 PMC4162951

[19] Finaret AB , Masters WA. Correcting for artifactual correlation between misreported month of birth and attained height-for-age reduces but does not eliminate measured vulnerability to season of birth in poorer countries. Am J Clin Nutr 2019; 110:485–97. 31179496 10.1093/ajcn/nqz111 PMC6669063

[20] Weber GW , Prossinger H, Seidler H. Height depends on month of birth. Nature 1998; 391:754–5. 9486642 10.1038/35781

[21] Almqvist C , Ekberg S, Rhedin S, et al Season of birth, childhood asthma and allergy in a nationwide cohort – mediation through lower respiratory infections. Clin Exp Allergy 2020; 50:222–30. 31782836 10.1111/cea.13542

[22] Golda A , Malek N, Dudek B, et al Infection with human coronavirus NL63 enhances streptococcal adherence to epithelial cells. J Gen Virol 2011; 92:1358–68. 21325482 10.1099/vir.0.028381-0 PMC3168281

[23] Atanasova K , Van Gucht S, Barbé F, et al Lipoteichoic acid from *Staphylococcus aureus* exacerbates respiratory disease in porcine respiratory coronavirus-infected pigs. Vet J 2011; 188:210–5. 20409735 10.1016/j.tvjl.2010.03.001 PMC2932768

[24] Chien YW , Klugman KP, Morens DM. Bacterial pathogens and death during the 1918 influenza pandemic. N Engl J Med 2009; 361:2582–3. 20032332 10.1056/NEJMc0908216

[25] Brealey JC , Chappell KJ, Galbraith S, et al *Streptococcus pneumoniae* colonization of the nasopharynx is associated with increased severity during respiratory syncytial virus infection in young children. Respirology 2018; 23:220–7. 28913912 10.1111/resp.13179 PMC7169064

