# Supplementary figures and images for: A Longitudinal Study of the Epidemiology of Seasonal Coronaviruses in an African Birth Cohort
Source: J Pediatric Infect Dis Soc. 2021 Feb 2;10(5):607–14. doi: 10.1093/jpids/piaa168 (PMC7928775; doi:10.1093/jpids/piaa168)

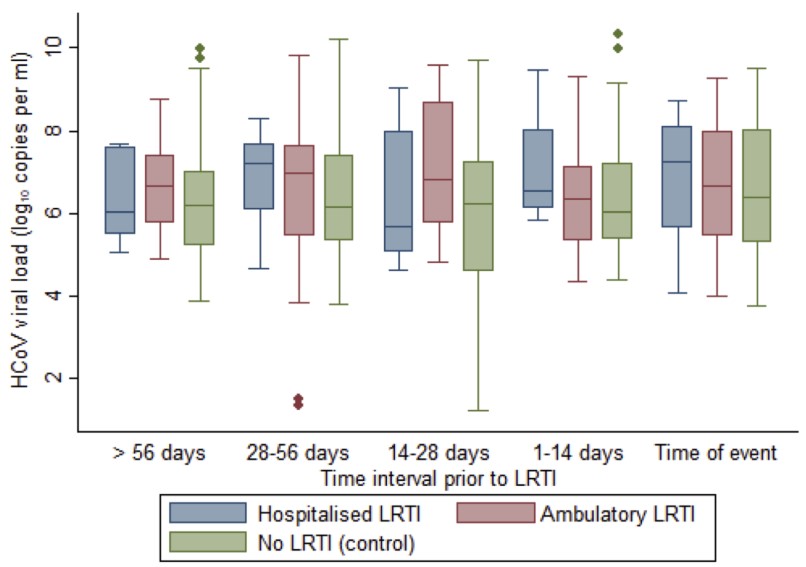

Supplement: piaa168_suppl_Supplementary_Figure_1 [file piaa168_suppl_supplementary_figure_1.jpeg]

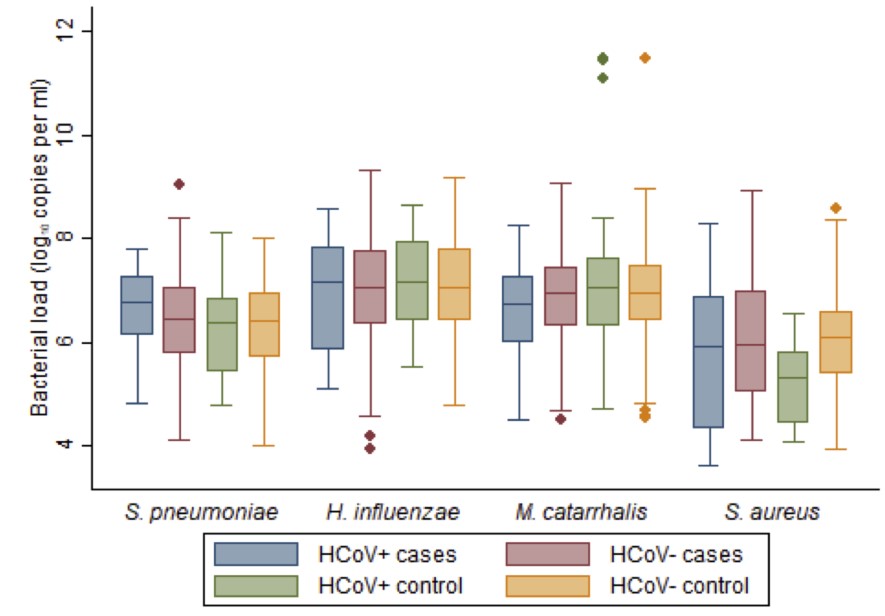

Supplement: piaa168_suppl_Supplementary_Figure_2 [file piaa168_suppl_supplementary_figure_2.jpeg]
